# Combining health insurance funds in a fragmented context: what kind of challenges should be considered?

**DOI:** 10.1186/s12913-019-4858-7

**Published:** 2020-01-08

**Authors:** Mohammad Bazyar, Arash Rashidian, Minoo Alipouri Sakha, Mohammad Reza Vaez Mahdavi, Leila Doshmangir

**Affiliations:** 1 Department of Health Promotion, Faculty of Health, Ilam University of Medical Sciences, Ilam, Iran; 2 Department of Health Management and Economics, School of Public Health, Tehran University of Medical Sciences, Tehran, Iran; 3 Department of Physiology, School of Medicine, Shahed University, Tehran, Iran; 4 Department of Health Policy and Management,Tabriz Health Services Management Research Center, Iranian Center of Excellence in Health Management, School of Management and Medical Informatics, Tabriz University of Medical Sciences, Tabriz, Iran; 5 Social Determinants of Health Research Center, Health Management and Safety Promotion Research Institute, Tabriz University of Medical Sciences, Tabriz, Iran

## Abstract

**Background:**

Iran’s Parliament passed a Law in 2010 to merge the existing health insurance schemes to boost risk pooling. Merging can be challenging as there are differences among health insurance schemes in various aspects. This qualitative prospective policy analysis aims to reveal key challenges and implementation barriers of the policy as introduced in Iran.

**Methods:**

A qualitative study of key informants and documentary review was conducted. Sixty-seven semi-structured face-to-face interviews were conducted, with key informants from relevant stakeholders. Purposive and snowball sampling techniques were used for selecting the interviewees. The related policy documents were also reviewed and analyzed to supplement interviews. Data analysis was conducted through an existing health financing World Bank framework.

**Results:**

This study demonstrated that for combining health insurance funds, operational challenges in the following areas should be taken into account: *financing mechanisms, population coverage, benefits package, provider engagement, organizational structure, health service delivery* and *operational processes*. It is also important to have adequate cogent reasons to *“the justification of the consolidation process”* in the given context. When moving towards combining health insurance funds, especially in countries with a purchaser-provider split, it is critical for policy makers to make sure that the health insurance system is aligned with the policies and *Stewardship* of the broader health care system.

**Conclusions:**

Implementation of major reforms in a health system with fragmented insurance schemes with different target populations, prepayment structures, benefit packages and history of development is inherently difficult, especially when different stakeholders have vetoing powers over the proposed reforms. Solving the differences and operational challenges in the main areas of health insurance system generated in this study may provide a platform for the designing and implementing merging process of social health insurance schemes in Iran and other countries with similar situations.

## Background

The risk for fragmentation in health financing through insurance systems exists when there is more than one insurance fund [1]. The degree of fragmentation in health insurance funds is a major concern in health financing and depends on the number and the size of risk pools and the amount of risk distribution between them [2–4]. Fragmented risk pools usually with different benefit packages for different groups of population lead to inequity in access to health care services. Fragmentation also hinders moving towards financial objective of universal health coverage as it reduces the potential degree of risk redistribution from a given amount of prepaid funds [5]. Combining prepaid funds together ideally in a single pool rather than keeping them in separate funds has been emphasized by international organizations such as World Bank [1] and World Health Organization (WHO) [6–8]. In countries with multiple pools, minimizing fragmentation by either merging them into a larger pool or by introducing an equalization fund to redistribute the risks between them should be considered as a policy to ensure that the people in the different pools are treated equally. Reducing fragmentation provides more financial protection from a given level of prepaid funds, which is the key objective of universal coverage [5, 8].

Consolidation of fragmented risk pools can be considered as a “Herculean task” bearing in mind the proponents (like governments or political parties to gain public support) and opponents (for instance the resistance of personnel and social health insurance (SHI) schemes to lose their autonomy due to disparity in the benefit packages and financial resources among them) [3]. Recent experience from countries like Vietnam, Turkey, South Korea and Thailand worked on reducing fragmentation in health insurance system over the last years shows different degrees of success, considering political challenges and operational obstacles in each country [9–12].

Two kinds of consolidation are common in the health care system: horizontal and vertical. “Vertical integration refers to consolidating (insurer and health care providers) under one organizational roof and common ownership all levels of care, from primary to tertiary care, and the facilities and staff necessary to provide this full spectrum of care”. Horizontal consolidation occurs when organizations in the same tier go through mergers or acquisitions. “A merger means two or more previously independent organizations consolidate into a single”. The latter kind of consolidation is common in the health system in the form of hospital mergers and insurer mergers. “Health insurance consolidation leads to substantial market power increases, cost savings and improved quality”.

### Health insurance system in Iran and the challenge of fragmentation

There are four main public health insurance organizations in the country. The Social Security Organization (SSO) is one of the largest health insurance organizations covering all the people employed in the formal private sector and their dependents. The Iran Health Insurance Organization (IHIO) has four sub-funds which provide health insurance for government employees and their dependents, rural residents, the self-employed (Iranians fund) and their dependents, and other sectors (such as students, some professional associations and so on). The Armed Forces Medical Services Insurance Organization provides health insurance for military personnel and their families. And finally Imam Khomeini Relief Foundation Health Insurance provides health insurance coverage for the poor [13–19].

There are considerable differences among them in terms of benefit packages, contribution rate, voluntary/obligatory coverage, per capita health care expenditures and using governmental financial subsidies. In addition to these main insurers, there are about 17 smaller institutional health insurance funds such as those offered by banks, private insurance companies, the Tehran Municipality, the National Broadcasting Organization, the Petroleum Industry Health Organization and so on which have launched health insurance coverage for their own employees and dependents outside of the main health insurance organizations [20, 21]. These better-off minor funds are usually small in population size. Enjoying high financial resources, these institutions provide generous health benefit packages for their beneficiaries, whilst not contributing to the whole risk pool [20].

Fragmentation in the Iranian health insurance financing model is rooted in incremental extension of insurance coverage over time due to contextual barriers such as low fiscal space. Fragmentation has directly or indirectly caused various problems for the health financing system like inequality in access to health care [17, 20]; inefficiency in health insurance system; high out-of-pocket expenditures [16, 17, 22]; low financial protection against health services for the insured persons [13, 23]; the high coinsurance rates; insurance coverage duplication [13, 16], lack of transparency and absence of reliable data and statistics for health insurance policy making [13].

In 2010, Iranian Parliament passed a law to amalgamate all the existing health insurance funds including 4 public health insurance schemes and 17 institutional funds (private health insurance companies providing supplementary services are not included) into the Medical Services Insurance Organization in order to create a new single health insurance organization named Iran Health Insurance Organization with the aim to centralize health insurance policy making in this organization [21].

### Objectives of the study

To the best of researchers’ knowledge there were no specific studies that objectively target the challenges and organizational considerations for the merging of health insurance funds. Hence this prospective policy analysis was done to answer this question that what kind of operational or structural challenges in what aspects of health insurance exist among health insurance schemes in Iran which may impede or affect the adoption of the policy or its successful implementation. The lessons derived from the present paper can be informative for policy makers from Iran and other countries, especially those with low- and middle-income, trying to merge existing health insurance schemes in order to strengthen risk pooling.

## Methods

### Interviews and documents

This is a qualitative study conducted in 2014–2015. To select study participants, four steps were followed: First, several local qualitative papers in the field of health financing and health insurance were studied and a list of relevant stakeholders including 7 key stakeholders was generated. Second, the articles of Consolidation of SHI Funds Law passed in the Fifth Economic, Social and Cultural Development Plan were studied and other relevant stakeholders were identified and added to the initial list (20 more stakeholders). The list was reviewed by members of the research team who were quite familiar with the context of health insurance system in Iran in order to identify and add other likely relevant missed stakeholders (6 more stakeholders). A purposeful sample of key informants with outstanding work and knowledge in the areas of health system, health insurance, and with relevant education, experiences and researches were selected to reflect all aspects of merging SHI schemes that are vague and not clearly understood. Other stakeholders and key informants were identified through snowball sampling. A total six other stakeholders were also identified based on information derived from the analysis of interviews and relevant documents. Various stakeholders were identified including Ministry of Health and Medical Education (MoHME); The Ministry of Cooperatives, Labor and Social Welfare (MoCLSW); Vice-Presidency for Strategic Planning and Supervision; Iran Health Insurance organization; SSO; Armed Forces Health Insurance; Imam Khomeini Relief Committee; the Parliament of the Islamic Republic of Iran (Majlis); The Medical Council of Islamic Republic of Iran; Health care providers; medical associations like Pharmacists Association, Association of General Practitioners, and Medical Laboratories Association; and finally minor health insurance institutes such as Petroleum Industry Health Organization, banks, Tehran Municipality, and National Broadcasting Organization.

To gather sufficient information and details needed to cover all aspects of policy questions, efforts were made to identify all relevant stakeholders occupying different positions on the subject. From each stakeholder, group or organization, sufficient samples were selected to obtain expert opinions and organizational positions. For this reason the number of interviewees in this study was greater than the standard for qualitative studies [24, 25]. Sixty face-to-face interviews were conducted. All interviews were conducted in the interviewees’ workplace as they were comfortable there. No one else was present for the interview besides the participant and researcher. Also seven further interviews were conducted with the health insurance staffs to obtain more detail information in some areas of health insurance. The minimum, maximum and average times of interviews were 10, 120 and about 50 min respectively. In seven cases, the interviews lasted for two to three sessions because interviewees were busy trying to finish the interview in one session, some of them were eager to give more information, and in some cases the interviewer had to refer again later for more details or to complete omitted information. Interviews lasted until data saturation was reached, a point at which “no additional data were being found” [26].

Documentary review was used as supplementary source of data collection. The principles of Five-Year National Economic, Social and Cultural Plans, 20 year national vision, national laws in the area of health insurance including Medical Health insurance Act (1994), TV programs, TV interviews, newspapers and proceedings of Iran Parliament regarding the Law of Consolidation were among the documents that were revised and analyzed.

### Conceptual framework

**Fig. 1:**
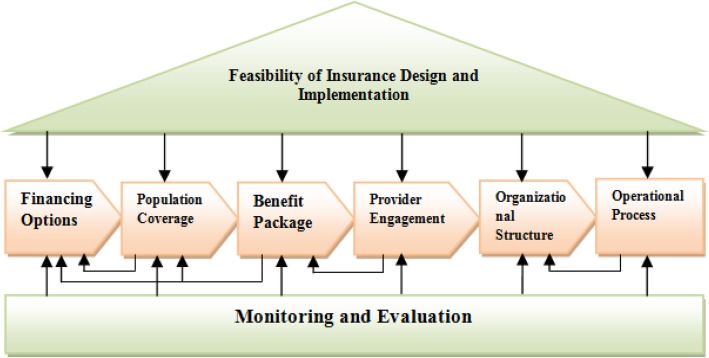
Design Elements for a Health Insurance Scheme

For the purpose of the study and to develop an appropriate conceptual framework to cover all aspects of the subject matter and to organize the findings, a vast fast review of literature about the models, tools and frameworks for analysis of health financing was conducted from which a relevant conceptual framework was derived from the World Bank (Fig. 1) [27]. The World Bank framework divides the steps in designing a health insurance organization into eight design elements: feasibility of insurance design, financing mechanisms, population coverage, benefits package, provider engagement, organizational structure, operational processes, and monitoring and evaluation. The purpose of World Bank framework is to help and provide policy makers in developing countries with a step by step guide to scale up existing health insurance funds or to design a health insurance plan from the beginning (not for merging). We used the framework for a new purpose, to classify the challenges of merging of health insurance schemes in Iran. This framework had been developed based on the lessons learnt and experiences gained from various environments all over the world. Covering the main aspects of health insurance, the members of the research team found it suitable and selected this framework for the study.

### Interview guide

A semi-structured interview guide was used to conduct the interviews (Additional file 1). The interview guide was designed to reflect the dimensions of the World Bank framework. In each dimension, challenges, differences or barriers that exist between health insurance schemes were examined. The following aspects were taken into account for merging: what requirements are needed, what are probable solutions for merging in each dimension, advantages of merging to health insurance system in particular and health system in general, what problems in the health system can be solved by merging, feasibility of merging health insurance schemes in Iran, actors and stakeholders involved in health insurance system and etc. The interview guide was developed based on the literature review (with a major emphasis on the World Bank framework), objectives of the study and existing situation of health insurance funds in Iran.

### Data collection

Depending on the experience and knowledge of the interviewees, purposeful and relevant questions were asked to get more detailed and practical information. For example if an interviewee had more information about the field of benefit package, alongside asking other questions in the interview guide, efforts were made to focus on and ask more probing questions about the benefit package in order to obtain a more detailed picture of this area that is influential on merging. At the start of each interview, by explaining the purpose of the study and ensuring the confidentiality of the content of the interviews and anonymity, interviews were taped with 2 sound recorders. In three cases, the participants did not allow voice-recording (they were afraid as the topic was political), therefore notes of the main points were taken down.

To collect documents, the list of related documents and their source for collection were identified. Websites of organizations including Majlis, MoHME, Iranian Medical Council, and Health Insurance organizations were reviewed and related in print documents were also collected in person.

### Data analysis

Content analysis using the ‘framework method’ was used to analyze the qualitative data. The framework method is a flexible tool which can be used in qualitative approaches that seek to generate themes. It is worth mentioning that this method can also be applied in deductive, inductive, or combined types of qualitative analysis [28]. The five-stage process of qualitative data analysis was done: understanding (familiarization), identifying a thematic framework (thematic), coding (indexing), charting and mapping and interpretation [29]. All the interviews were done, transcribed and initially indexed by one author (MB). The analysis of interviews began with the initial eight-element framework of World Bank (deductive approach). At the same time the authors remained flexible to develop and add new elements to the initial framework according to the emerging data (inductive approach). The main themes of the framework were extracted by using MAXQDA software. Two authors discussed the themes and identified items in in-depth discussions and used the deliberations to update the thematic framework. The main themes of initial framework were discussed four times in meetings among the members of the research team. The main themes were revised several times, eventually resulting in eleven themes being developed. The 11 main themes were showcased in a focus group discussion of experts that was aimed to find solutions for the challenges facing the implementation of the merging policy (which was not the aim of this article). It’s worth mentioning that transcripts were not returned to participants for comment and correction but during the interview more details were asked for clarification whenever it was necessary.

## Results

**Fig. 2:**
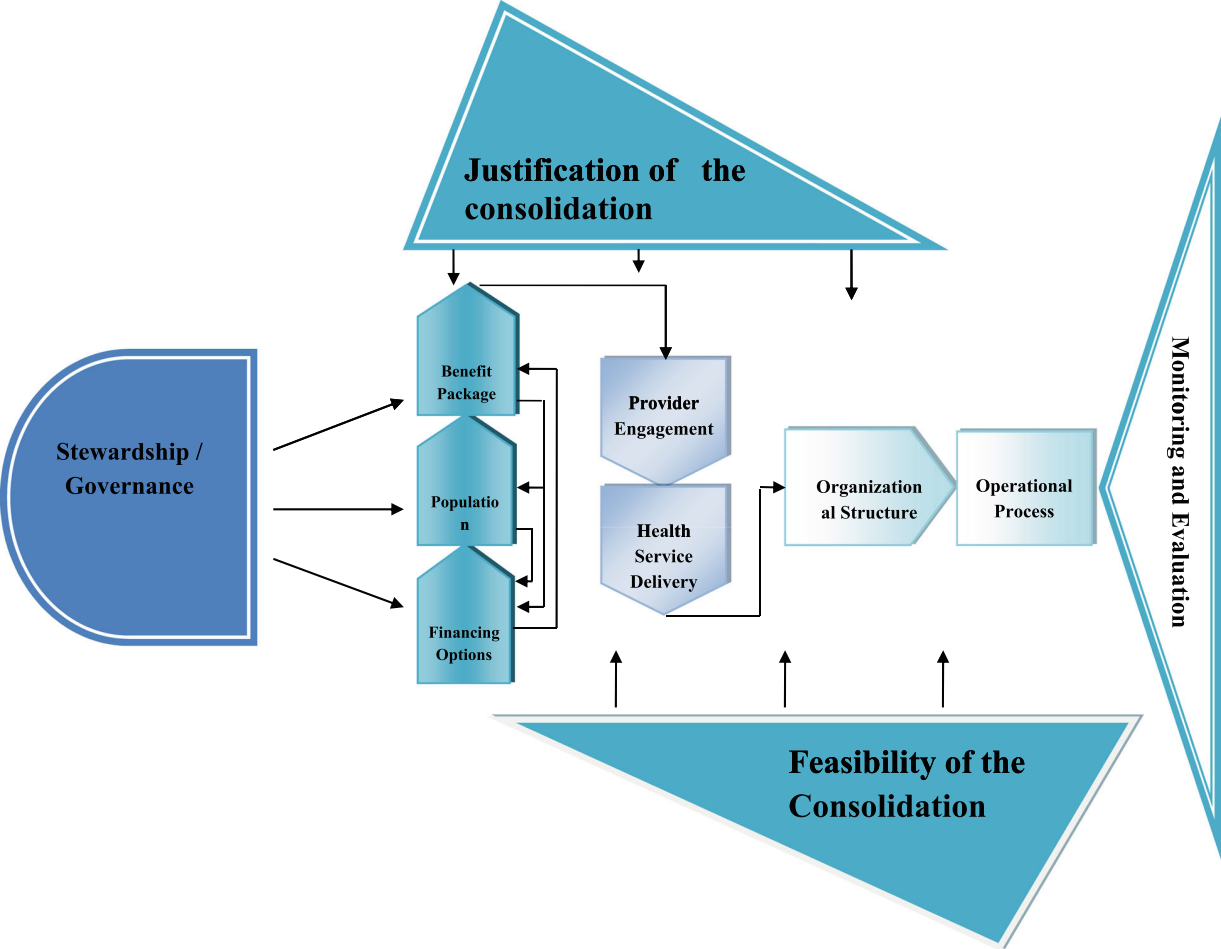
The proposed framework for merger of existing health insurance organizations in a middle-income country

Out of 67 interviewees, 62 were male and five were female; reflecting the composition of female representation in high level positions in insurance organizations and relevant departments in the ministry of health. Participants’ age ranged from 33 to 80 years. Facing a complex health insurance system with a long history in Iran, the selected framework (eight-element World Bank framework) was not comprehensive enough to cover all aspects of the complexity of merging. Based on emerging findings of this study, three new elements including “Justification of the consolidation process; the explicit definition of the policy objectives”, “Stewardship” and “Health Service Delivery” were generated through qualitative data and added to the initial framework. Therefore the finalized framework developed in this study includes 11 elements which is depicted in Fig. 2 (fish-shaped framework). The study proposes that all the eleven items should be considered within any plan to consolidate and merge existing health insurance determine organizations and in each element the main focus was to mention the key challenges and operational barriers which should be solved. It is worth to mention that there is no linear relationship between all 11 elements but we tried to provide a logical order for the elements of the framework. The justification of merger and the feasibility of merger should be determined before the merger and should be considered throughout the merger process and they were chosen to form the figs of the fish. The element of stewardship forms the head of the fish model as it should lead and control the rest of elements. There is a close relationship between three elements of financing, population features and benefit package in health insurance and policy makers should focus on these elements at the early stages of merging. After that policy makers should think about the differences between health insurance schemes in terms of how to contract with health care providers and how to provide health services for beneficiaries. We believed that changing the organizational structures and operational processes of health insurance schemes should be addressed later on in the final phases of merger. And finally all stages and the whole process of merger should be monitored and the short term and long term consequences of merger must be evaluated to see whether the expected effects of merger have been materialized after merging.

### Justification of the consolidation process

Consolidation is a tough political process and will face many political challenges. So, identification of the policy objectives of consolidation is critical to justify the implementation of merging and also reduces the resistance of opposing opponents based on convincing scientific evidence.

According to the participants, the main reasons for merging the existing SHI schemes were as follows: reducing inequity in accessibility to the health care and health benefits packages among different groups of population; reaching universal health coverage at its three dimensions, and failure of previous laws to solve the problems of fragmentation in SHI funds without touching their structural and organizational autonomy. According to the proceedings of the IR Iran Parliament, one of the public representatives directly involved in passing the Consolidation Law in the Parliament said:

*“ … We proclaim that we work for equity but at the same time we have spread special colorful tables for some groups of population while some other groups do not get anything … inequity makes people feel segregated … it doesn’t matter whether the government has budget or not, it is important to provide the same benefit packages for ourselves as poor people have (for all) … ”*

### Stewardship

The second item of the developed conceptual framework is stewardship. In 2004, a purchaser-provider split occurred in the Iranian health system in order to centralize policy making and regulating for all SHI schemes in the High Council of Health Insurance (HCHI) under the Ministry of Co-operation, Labor and Social Welfare (MoCLSW), and to provide the opportunity for the health insurance schemes to find their real function and to purchase good quality services on behalf of beneficiaries from the MoHME as the main provider [30]. From then on, policy making of the health system and the SHI schemes has been performed separately by MoHME and MoCLSW. After this separation, due to lack of cooperation and coordination between the two ministries, MoHME faced new challenges for expected stewardship and implementing health reforms without control on financial resources. According to the participants, the stewardship should not be a matter of organizational fragmentation or integration among health insurance schemes or between ministries of MoHME and MoCLSW. But past experience of health system in Iran indicates that institutional fragmentation both in ministries and health insurance schemes levels has led to fragmentation in stewardship of health system in Iran. Thus when designing a health insurance system, especially in countries with a purchaser-provider split, it is critical for policy makers to make sure that the health insurance system is aligned with the broader health care system and its policies.

*“In practice, health insurance schemes are not accountable to the Ministry of Health as the only body responsible for stewardship of the health system and are not aligned with its policies. They follow their own policies and state that” they are accountable to* MoCLSW *not the Ministry of Health”...” (A governmental policy maker from MoHME)*

Despite the establishment of the High Council of Health Insurance (HCHI) in 1994 as an entity responsible for regulating of SHI schemes, fragmentation in the health insurance system caused each health insurance scheme interpret and implement policies declared by HCHI differently in practice. Multiple SHI schemes with financial and managerial autonomy provide the opportunity to make policies separately, which also leads to fragmentation in the health insurance system stewardship. Creating a single insurance organization would centralize stewardship for the health insurance system.

*“ … currently each health insurance company makes its own policies separately; this individualism in decision making would be automatically removed by merging, and this is a great success … ” (Senior expert from Pharmacists Association) “ … The objective of merging is to centralize policy making for the health insurance system in one central organization and to prevent health insurance stewardship from being fragmented … ” (A policy maker from IHIO)*

### Feasibility

Merging risk pools especially in developing countries is a challenging issue. According to the interviewees, feasibility of merging must be considered in five dimensions including technical and managerial, economical, socio-cultural, political and legislative.

In Iran, the main public SHI schemes are governmental and they rely mainly on the government budget for funding. Presently they are struggling with some financial problems including chronic inadequate financial resources [13, 23], considerable share of population working in informal economy with no regular wage [16, 20], about 10% of whole population without health insurance coverage [20], and high out of pocket health care expenditures (around 54% of total health expenditures) which can refer to not effective health insurance system as one of the contributing reasons [31]. Considering these problems, considerable financial attention is required when planning to create a single national health insurance. It is also important address the question to what extent does the successful implementation of consolidation rely on the economic status of the country and how committed are the officials to allocating more budget for a financially sustainable single scheme.

*“ … this year the government doubled the budget of Rural Fund, but we are concerned about the financial sustainability, we can’t define the benefit package based on budget which we aren’t sure about it” (An expert from IHIO)*

### Health service delivery

This dimension refers to how SHI schemes provide health services for their beneficiaries, what are the probable differences between them, and how these differences should be approached while merging multiple SHI schemes. Some health insurance schemes have established their own health facilities and hospitals to deliver free of charge health services for their beneficiaries while other SHI schemes have not. SHI schemes pay higher medical tariffs for reimbursement to their medical centers and pay higher payment to the medical personnel working in their centers. Another serious difference is that some health insurance schemes have launched referral systems and family physicians for managing funds, and the beneficiaries could access health services only by following the strict regulations of referral system while other insured groups can access specialized services freely.

*“ … Another challenge for merging is the referral system. Currently, following the referral system is not obligatory in the health system, but we have launched a referral system within our insurance for beneficiaries and tried to improve and promote it … ” (An expert from Petroleum Industry Health Organization)*

*“ … we have hospitals and clinics in some places which are not profitable, but we have to keep those facilities despite high operational costs because of health needs of our beneficiaries. With consolidation, IHIO will reimburse these facilities based on persons who live in those places and use health services … ” (A senior manager from Petroleum Industry Health Organization)*

The effect of consolidation on the quality and quantity of heath care services delivered by the health system should also be considered in the service delivery dimension. Where health insurance companies as the purchaser of heath care services do not have enough control and supervision on importing health technology equipment, pharmaceuticals, increasing total health care expenditures, and the amount of services delivered by providers or consumed by beneficiaries, consolidation might not have tangible effects on the fund management.

*“ … the Iranian health system is like a container with a wide opening and a lot of holes in the bottom, no matter how much money you inject into it, it will be drained away soon … ” (An expert from Armed Forces Health Insurance)*

### Financing

Fragmentation of the health insurance funds and insurance schemes financial autonomy in Iran has caused great disparities in the revenue collection processes. There are differences in the contribution rate, wage ceiling and various percentages of wages for premium deduction, fund resources, user charges, and per capita tax subsidy. Each of these disparities is a source of resistance against merging that needs to be addressed carefully and revised in a way to facilitate the process of consolidation and to enhance financial sustainability.

*“ … the first requirement for merging health insurance schemes is revising and defining an acceptable contribution rate for all schemes … for example, in SSO, the contribution rate is 9% of salary up to 7-fold of the minimum wage, while for government employees, this rate was 5-% previously and is 6-% now but only up to 2-fold of the minimum wage. Rural population fund, one of the largest health insurance schemes with about 23 million people is completely free and the contribution rate is 6% of the minimum wage (paid by the government). For some population groups the contribution rate is fixed and for banks and some minor funds the contribution rate is several-times more than the public insurance rate about 13% with no ceiling … putting all these disparities together, you see policy makers can't formulate the same policy for all … ” (A senior manager from IHIO)*

### Population coverage

Developing health insurance coverage for different types of population over time has led to almost homogenous populations for each insurance scheme with almost the same socioeconomic status, risk of getting sick, need of health services, and sometimes demographic characteristics. These differences among risk pools and even special expectations and demands of some population groups have forced health insurance schemes to change and modify the basic benefit package to meet the needs accordingly. These differences should be considered carefully while developing a unified basic benefit package for the single scheme.

*“ … you can't provide the same health benefit package for the petroleum industry employees, government employees, people living in urban and rural areas, or people covered by Imam Khomeini foundation; for this reason, a single health insurance scheme must be so flexible that could meet all needs and requirements of all population groups or they (SHI schemes) must be individually organized … ” (A policy maker from Parliament)*

Various population sizes from less than 50,000 enrollees to about 37 million enrollees and voluntary or obligatory enrolment are among other differences between schemes that might cause some operational challenges for consolidation. More than 90% of the population is under the coverage of two largest schemes (SSO and IHIO) with almost the same health benefit packages. The rest of the population is covered by other insurance schemes with generous health coverage. Inequity between these two mentioned segments of population is more severe. These different population sizes are important because some ideas of interviewees about the merging process were based on the population size. There were pros and cons whether begin merging from small or large schemes. Some interviewees for several reasons believed that it is better to start from small schemes such as those 17 minor well-resourced schemes as they don’t work under the regulations of HCHI and enjoying financial resources and generous benefit package while have no contribution to the basic health insurance system, they purchase health services from private health sector at the higher prices compared with public SHI schemes. So by merging these small funds together under the umbrella of the existing public SHI schemes such as IHIO it is possible to reduce the number of risk pools with more balanced population sizes and solve the problem of fragmentation to some extent. For example, one interviewee said:

*“ … if they want to merge all insurance schemes they should begin with large schemes, it is true that we have various health insurance funds but we should consider their population size, as well … for example, petroleum health insurance fund has about 600,000 enrollees, which is not large and is not considerable in comparison with the whole population” (A senior manager from Petroleum Industry Health Organization)*

*“about 35 million people are under the coverage of IHIO and almost the same population size is covered by SSO. So the merging would be much easier, if the conditions for providing the same benefit package for these two schemes are ready” (A senior manager from IHIO, TV interview)*

### Benefit package

In Iran, four main basic health insurance schemes are regulated under the supervision of HCHI and they should provide the same basic benefit package according to health insurance laws. Other minor better-off schemes are not under the supervision of HCHI and provide almost all health services available for enrollees with few exceptions. Because of fragmented risk pools, health insurance schemes are financially, managerially, and structurally autonomous. The autonomy, non-comprehensive basic benefit package, special demands and medical needs of different groups of population have forced health insurers to provide more health services for their beneficiaries than the basic benefit package. Improving equity and providing the same benefit package for all is one of the prerequisites of creating a single payer. In defining a new basic benefit package for consolidation, the differences mentioned earlier should be considered.

*“ … Insurance schemes have different price lists for drugs under coverage and they don't react to the changes in drug prices in the market at the same time which increases inequality among different groups of enrollees … ” (An expert from SSO)*

One strong source of resistance against merging is the health insurance schemes with generous benefit packages, especially in Iran where other schemes must be merged into IHIO with the weakest benefit package based on the law. Their resistance originates from previous perception of inadequate benefit package, low performance, and low level of contribution rate in IHIO.

*“ … minor better-off schemes can't tell their employees that you will get a health benefit like a government employee from tomorrow, sorry to say that but the lowest possible. This obviously would create social problems … ” (An expert from IHIO)*

### Engagement, selection, and payment of health care providers

In countries like Iran where a mixture of providers is available, one important difference among SHI schemes is the extent of accessibility that beneficiaries have to all kinds of providers. Some schemes mainly focus on public hospitals to purchase medical services for their beneficiaries but other scheme’s beneficiaries can freely access health services from their own medical facilities, whether private or public.

*“ … Some health insurance schemes have a high capitation premium but I can say with no doubt that about 60 % of this fund is driven by private centers; we don’t purchase medical services from the private sector, we say we buy it [medical services] from public centers but completely free for our enrollees. Actually, we can somehow call it the management of financial resources … ” (An expert from Imam Khomeini Foundation)*

In Iran the main payment method used by health insurance schemes is fee for service. However, some insurance funds like rural household fund use the capitation payment for reimbursing providers. Using different tariffs for payments is another main difference between health insurance schemes. SSO and IHIO reimburse all providers including private, public, teaching hospitals based on public medical tariffs. Other insurance funds with high financial resources pay private providers based on private tariffs (which provide a great deal of outpatient and inpatient health services) and provide better financial protection for their enrollees. Even those schemes with their own medical facilities like SSO may pay higher tariffs for their facilities and follow different policies for reimbursing. All these differences will pose additional challenges for the consolidation of health insurance funds.

*“ … IHIO wants to treat our medical centers (SSO) based on public tariffs it pays to other medical centers?!...” (An expert from SSO)*

As a result of fragmented health insurance system in Iran, each scheme follows different regulations for contraction with the providers.

### Organizational structure

An important structural issue regarding merging SHI schemes is to create an entirely new organization with new identity and mission for insurance administration or using existing health insurance structures. Establishing a new organization increases administrative expenses and is not justifiable in terms of efficiency. If one of the existing health insurance organizations is supposed to be the center of merging, an appropriate selection would have a great effect on reducing resistance from other health insurance schemes against merging. Creating and introducing a new autonomous impartial health insurance organization as a national agency for merging other insurance schemes into it will cause less resistance from other organizations.

*“ … although the Law says that they [health insurance schemes] must be merged into IHIO, there is no willingness; for example, SSO says why IHIO should not be merged into Social Security Organization?! The rest of schemes have similar opinions … ” (An expert from IHIO)*

Ownership of organization (governmental, public, private, non-governmental), the size of the organization in terms of population coverage, geographical coverage, capacity of administrative structure, financial capability, and benefit package, performance, experience and precedence are some criteria that could be used when choosing one of the existing organizations for the purpose of consolidation.

According to the interviews and TV programs *(6 sessions of a TV program called Nabz were about the Consolidation Law)* following reasons were mentioned for selecting IHIO to merge other health insurance schemes under its umbrella: *being governmental, working specifically as a health insurer, focusing on its role as a purchaser without owning medical centers, (A Senior expert from Vice-Presidency for Strategic Planning and Supervision), having administrative structure in place all over the country (A governmental policy maker from MoHME).*

Currently, each insurance scheme has its own administrative body and board of directors, but a single health insurance means there would be one board of directors. Identifying and selecting new members for the administration of the new health insurance organization would be a tough political decision. One of the sources of resistance against merging results from top managers concerns their organizational positions. Employees in different insurance schemes work with different salaries and use different instructions for similar activities.

*“ … merging would have the greatest impact on the top managers. For example, SSO and IHIO have their own general directors in each province. Merging several organizations means two general directors become one and 10 deputy directors become 5 … ” (An expert from IHIO4)*

### Operational process

Among other operational processes like expenditure tracking system, cost management, contract management, marketing and communication, beneficiary identification and enrollment, collection of financial contributions, claims administration, the following financial issues are important for successful consolidation: establishing precise financial flows and an expenditure tracking system to ensure collecting of adequate financial resources for covering expected costs, ensuring maintenance of financial equilibrium for the national single health insurance, and ensuring transparency and accountability in financial flows to provide precise financial reports. Differences in operational processes are important but are less difficult to address. Health insurance schemes follow different procedures full of details for reviewing claims. Although solving these details among schemes is not very hard but formulating a new process for claims administration is an operational challenge for creating a single scheme. One interviewee mentioned the importance of accountability of financial processes:

*“ … years ago when ministry of health was responsible for providing health services for SSO beneficiaries, there was no financial transparency … The parliament obligated ministry of health for several years to report its financial performance, but it did not!! ministry of health didn’t report about how and where it spent the SSO funds … ” (An senior expert from SSO)*

*“ … we cover those people who are poor, disabled, sick … but SSO does screening tests before covering applicants, they don’t cover pre-existing diseases, they reject high risk applicants … ”(An expert from Imam Khomeini Foundation)*

### Monitoring and evaluation

The purpose of this element is to monitor and evaluate the quality of implementation process of consolidation. For effective implementation, all concerns and issues mentioned in previous elements should be considered carefully. Given the operational and executive challenges of merging, it is necessary to constantly compare the quality of ongoing implementation process with the designed plan.

*“ … major policies like merging in Iran need specific considerations for successful implementation like driving and deterrent factors … it requires corrective actions in structures, financing, regulations, culture, processes etc in long term to make sure that required changes are made in the implementation process of merging … it needs proper policies for rewarding and punishing … ” (senior academician)*

The reasons behind consolidation could be used as direct outcomes to evaluate the success of consolidation in achieving them. It is important to set measurable indicators for various dimensions of health insurance like management performance, financial performance, population coverage, benefit package, etc. to measure if the merging has brought about any improvements.

## Discussion

The 11-element framework introduced in this study is more useful for countries with multiple social health insurance schemes in place, which try to merge them in order to strengthen risk pooling across the health insurance system.

### Why merging? Is merging enough?

The current study showed that implementation of merging in Iran was not an easy decision considering the fact that it faced strong oppositions from its opponents. Resistance of top managers of health insurance schemes to lose organizational autonomy and try to avoid transparency in organizational statistics, information, and activities, concerns about paying more contribution rates after merging, resistance of better-off SHI schemes to share advantages with less privileged groups as a result of merging, concerns of personnel and top managers about their organizational and financial status are among some of the reasons why opponents are against the merger [32]. Therefore, there should be adequate, acceptable and convincing advantages for merging. The necessity of merging is still a contentious issue among the proponents and opponents in Iran. This controversy between experts has been one of the main obstacles for the implementation of consolidation.

Most experts mainly from MoCLSW insist on supply side problems within the Ministry of Health of which solving them should precede the consolidation process. Improving provision of health care in rural areas and smaller towns, expanding the family physician system to urban areas, improving the referral system, establishing a comprehensive electronic system for the medical records, furthering the use of evidence-based clinical guidelines, moving away from fee for service provider payment approaches, and controlling overall health care expenditures, were some of the main problems in the Iranian health system considered by the health insurance experts as more important than the fragmentation in the health insurance system which should be addressed first [19]. In other countries like Turkey, South Korea, Indonesia, and Thailand, among many other reasons, reducing inequity in access to health care services was the core reason to move towards reducing fragmentation in the health insurance system [ 9, 10, 33, 34].

From the feasibility perspective, prerequisites in the political, economic, socio-cultural, legislative and technical dimensions are important. In developing countries, more attention should be paid to the availability of infrastructures, skilled labor-force and above all, the fiscal space of government to provide sustainable financial support for the single scheme, especially in long term. Creating a single insurance means that critical decisions in the health insurance area, such as adjusting contribution rates or modifying health benefit package that were previously made at local levels become national issues. It makes the process of adjusting contributions more difficult and can lead to a fiscal deficit in the case of increase in healthcare expenditures. Experiencing the financial instability after merging, as observed in South Korea, requires more financial support from the government [35].

### Merging health insurance funds in Iran and path dependency approach

The findings of the study implied that a part of challenges facing merger health insurance schemes in Iran are rooted in the past decisions. In other words, past policy decisions and the path taken by actors until now act as structures that can limit or shape current policy options and can have a profound limiting influence on future policy-makers. So “path dependency approach” was applied to explain some parts of complexities facing consolidation process in the Iranian health insurance system. As Wilsford noted “actors are hemmed in by existing institutions and structures that channel them along established policy paths [10]. Simply this theory suggests that history is important [36, 37].

As Greener (2002) argues, when using the path dependency approach to analyze social and political processes, efforts should be made to differentiate between the two kinds of factors based on their effects on the change process: those factors with relatively permanent features of the policy environment and those more transitory factors. Both factors are necessary for a policy change to occur. The permanent factors which refer to structural factors require modification for the introduction of new policies [38]. Some of the political and executive challenges in different elements of our conceptual framework originate from factors with almost structural features. Facing such policies dependent strongly to the previous path in the following aspects such as legislative feasibility, structure, service delivery, and benefit package makes it more difficult to follow a new path radically different from the established path [39] even when it is generally accepted that those old policies may no longer be efficient [37]. For instance, it is not easy to change some laws in the Iranian health insurance policy due to legislative legacies and a strong historical path in these areas dating back to 1975 [39]. For example, since 1975, SSO has faced many changes in terms of providing health care services for its beneficiaries over these years. At the beginning, other organizations were responsible to treat SSO beneficiaries and SSO agreed to entrust premiums to them. This responsibility was transferred to MoHME in 1985. Over those years, SSO officials were worried about spending SSO’s premiums elsewhere rather than treatment of their beneficiaries. Lack of transparency in financial flows and lack of responsiveness of MoHME towards SSO forced the Iranian Parliament to pass the Obligation law in 1989. According to this law, SSO was obliged to take the responsibility of providing health services directly. As a result hospitals and medical health centers running by SSO were extended to provide free of charge health services for the beneficiaries. Now this historical path has become a strong legislative obstacle for consolidation of the existing schemes in Iran “which inhibits the introduction of ‘better’ or perhaps more rational policies or new organizational forms” [37, 40]. Despite increasing health care expenditures in SSO’ health centers resulting from moral hazard due to free health services, it is politically and legally difficult to introduce some kinds of cost sharing for the workers in these centers to restrain expenditures. For once, efforts to abolish free health services were rejected because it was considered as being against the Obligation Law. Creating a single health insurance in Iran with the same benefit package for all to reduce inequity in health care utilization means the current differences between benefit packages of SHI schemes should be abolished. It means that workers are no longer entitled to free health care services in SSO’ hospitals and clinics which is hard in practice as it is against Obligation Law. Another possibility is to provide free health care in public hospitals at least for IHIO and SSO beneficiaries to reduce the resistance of workers, although this policy seems expensive and inapplicable. Similarly, citizens accustomed to free and generous healthcare services in well-resourced minor schemes have made it more challenging to determine a more equitable benefits package for all. According to these kinds of path dependent challenges, it is advisable to follow more conservative policies and keep the differences between benefit packages at the beginning of the merging. It implies that while creating a single payer with the same basic benefit package for all, other well-resourced schemes will be allowed to provide extra services for their beneficiaries that they are used to or SSO should continue to deliver free health services in its health facilities for the workers as usual. Path dependent policies in other countries show the same experience. The strong Bismarckian tradition in the health insurance system in the Czech Republic and being accustomed to free health care services under the communist regime restrained policy makers to introduce more market-oriented health insurance system after the collapse of communism in 1989 [39].

Changing organizational structures is more difficult. Current institutional legacy and pre-existing buildings of SHI schemes in Iran is not easy to change to move towards creating a single health scheme. For example as a result of the Obligation Law, a new department called Health Deputy was formed within the SSO aiming to supervise the quality of health care delivery to the workers. That is why interviewees believed that structural merging of SHI schemes into IHIO and reducing the parallel organizational structures of health insurance schemes and also the number of employees and managers should be the final step of merging. To evade the path dependent institutional features of current SHI schemes in Iran and to help create a single payer with new mission, some of interviewees suggested establishing a new independent organization for merging to reduce the resistance of the existing schemes (although this will increase the administrative costs). This suggestion was based on the conviction that using pre-existing institutions to bring new approach to the health insurance system is hard. Some other interviewees suggested a conservative direction for structural merging, extending the current structure of IHIO (currently it has four separate individual sub-funds for different segments of population with the same benefit package under a united oversight organization) to the other groups of population.

### Study strengths and limitations

As literature about the experiences of merging health insurance funds in other countries was not rich and we found no study to address this issue directly, we had some limitation in addressing the shortcomings of our research in the context of peer-reviewed work derived from international context. While this limitation might enhance the importance and value of this study, at the same time it suggests that the findings should be interpreted with caution. Lessons and models discussed here can be enlightening for other countries. This study was conducted after passing the Consolidation Law in Iran when the government was mandated to implement the law. Hence it provides a natural policy environment in which the expectations and challenges of the stakeholders in implementation of the law, as well as their conflicting interests, could be better captured in the study. At the same time some of the interviewees may have constrained in expressing their opinions due to political considerations and their day-to-day responsibilities and institutional positions. By following heterogeneous sampling and getting opinions of different actors, both opposing and supporting the policy, as well as providing ample opportunities to the interviewees to express their deep understandings of the context, we tried to control this potential source of bias.

## Conclusion

As discussed throughout the article, the experience of Iran shows that it is not easy to reduce fragmentation in a disintegrated health financing system due to many challenging operational obstacles existing in different areas of the health insurance system. Policy makers and implementers should consider, manage and solve simultaneously the differences and barriers which exist in the main dimensions of health insurance including the benefit package, financing, population coverage, institutional features, the delivery of health care services, and engaging with health care providers mentioned throughout the article. These areas are intertwined which makes the process of merging more difficult, because it needs to manipulate several aspects of health insurance at the same time. For instance, changing the benefit package for all requires the changes in the current way of setting premiums, or pooling the premiums of different groups needs defining new kinds of structural forms. The path dependency approach illuminated a part of resistance of key actors against creating a national health insurance scheme in Iran and reasons behind it. It helped us to learn that as multiple health insurance schemes were created over time and as some kinds of path dependent policies were formed; it has become much more difficult to move away from the old paths. This causes additional operational problems for reducing risk pools in the future. So according to the experience of Iran, it is advisable to think about merging health insurance funds together and reducing fragmentation at the early stages of health insurance system when health insurance schemes are still young to avoid forming these kinds of path dependency problems. Across all systems, major reforms are less common. They are usually quite difficult, although not impossible. Implementation of major reforms, such as merging health insurance schemes structurally, in a fragmented path-dependent system with quite numerous veto-ridden opposed actors is more difficult [40, 41].

The authors believe that this framework could provide policy makers and researchers from other contexts with a comprehensive model to address and analyze the concept of consolidation of health insurance schemes with a better insight. The challenges mentioned in Iran for merging in each element of the framework can be informative for policy makers in other countries and remind them of the same or relevant issues in their own context. As three new dimensions were added to the WB framework based on the characteristics of health insurance system in Iran which should be considered for merger, it indicates that the framework likely needs development by researchers from other countries to cover other issues which may matter while approaching merging health insurance schemes in their context. As the merger of health insurance schemes in Iran has not implemented yet, it is impossible to see whether the effects of the challenges materialized after the merging of the insurance schemes.

## Supplementary information

**Additional file 1.** Interview guide for merging health insurance schemes in Iran.

## Data Availability

All raw data and also the file of thesis have been prepared in Persian (not English). But the corresponding author will gladly provide any supporting materials upon request.
