# Correction to: Combining health insurance funds in a fragmented context: what kind of challenges should be considered?

**DOI:** 10.1186/s12913-020-5052-7

**Published:** 2020-03-19

**Authors:** Mohammad Bazyar, Arash Rashidian, Minoo Alipouri Sakha, Mohammad Reza Vaez Mahdavi, Leila Doshmangir

**Affiliations:** 1 Department of Health Promotion, Faculty of Health, Ilam University of Medical Sciences, Ilam, Iran; 2 Department of Health Management and Economics, School of Public Health, Tehran University of Medical Sciences, Tehran, Iran; 3 Director of Information, Evidence and Research Department, World Health Organization Regional Office for the Eastern Mediterranean, Cairo, Egypt; 4 Department of Physiology, School of Medicine, Shahed University, Tehran, Iran; 5 Department of Health Policy and Management,Tabriz Health Services Management Research Center, Iranian Center of Excellence in Health Management, School of Management and Medical Informatics, Tabriz University of Medical Sciences, Tabriz, Iran; 6 Social Determinants of Health Research Center, Health Management and Safety Promotion Research Institute, Tabriz University of Medical Sciences, Tabriz, Iran

**Correction to: BMC Health Serv Res**

**https://doi.org/10.1186/s12913-019-4858-7**

In the original publication of this article [1], there are two corrections:

1. The Fig. 1 is mistakenly replaced by Fig. 2 in the pdf version, so that the Fig. 1 and Fig. 2 are the same. This error is caused by a typesetting mistake. The corrected Fig. 1 is shown below:

**Fig. 1:**
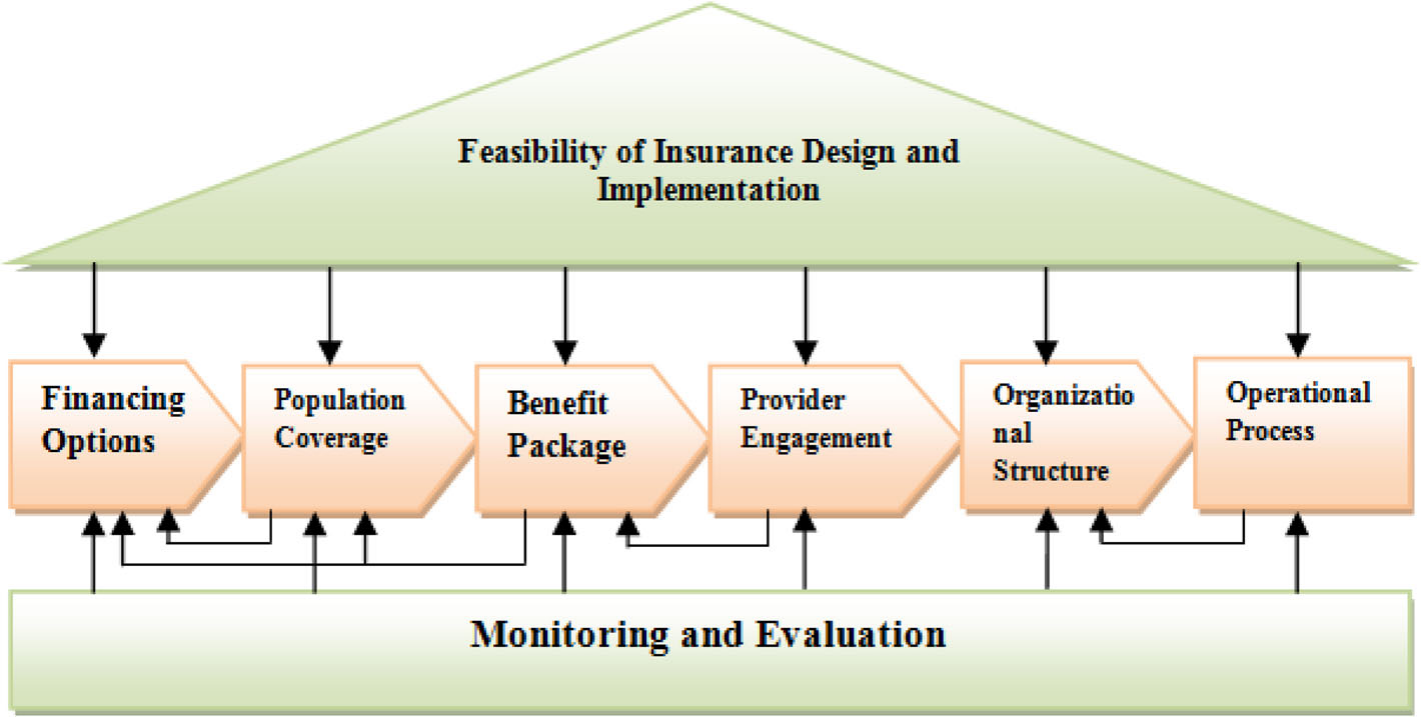
Design Elements for a Health Insurance Scheme

2. Arash Rashidian has another affiliation, which was missing in the original article. The author details are revised as below:

Mohammad Bazyar^1*^, Arash Rashidian^2,3^, Minoo Alipouri Sakha^2^, Mohammad Reza Vaez Mahdavi^4^ and Leila Doshmangir^5,6^

^1^ Department of Health Promotion, Faculty of Health, Ilam University of Medical Sciences, Ilam, Iran.

^2^ Department of Health Management and Economics, School of Public Health, Tehran University of Medical Sciences, Tehran, Iran.

^3^ Director of Information, Evidence and Research Department, World Health Organization Regional Office for the Eastern Mediterranean, Cairo, Egypt.

^4^ Department of Physiology, School of Medicine, Shahed University, Tehran, Iran.

^5^ Department of Health Policy and Management, Tabriz Health Services Management Research Center, Iranian Center of Excellence in Health Management, School of Management and Medical Informatics, Tabriz University of Medical Sciences, Tabriz, Iran.

^6^ Social Determinants of Health Research Center, Health Management and Safety Promotion Research Institute, Tabriz University of Medical Sciences, Tabriz Iran.

The original article has been updated.
