# Supplementary material for: Combining health insurance funds in a fragmented context: what kind of challenges should be considered?
Source: BMC Health Serv Res. 2020 Jan 8;20:26. doi: 10.1186/s12913-019-4858-7 (PMC6950996; doi:10.1186/s12913-019-4858-7)
Supplement: Supplementary file 1 — Additional file 1. Interview guide for merging health insurance schemes in Iran. [file 12913_2019_4858_MOESM1_ESM.docx]

**For interviewer:**

Introduce the aim of the study.

Make sure to get consent of the interviewees to participate and record their voice and for direct quotes from their interviews to be published in this manuscript with protection of their anonymity and confidentiality.

**For interviewees:**

In your opinion why the law of merging health insurance funds was passed in Iran? to solve what problems? please explain.

To facilitate merging, what kinds of differences or challenges exist between insurance funds in the following areas which should be addressed? financing? benefit package? population coverage? structure? operational processes? please explain.

To sole operational problems in the above areas, what solutions do you suggest? please explain.

What kinds of problems in the health insurance system particularly and in the health system generally will be solved by merging? Explain the advantages of merging. please explain.

What kinds of difficulties or challenges may happen in the health insurance system particularly and in the health system generally as a result of merging which should be considered? please explain.

Do you see the current situation in the health system and health insurance in Iran ready for the merging? Is it feasible to merge insurance funds together now? please explain.

Who are the main stakeholders of merging? explain their position (for or against)? and why?

What stages and steps do you suggest to be followed to merge insurance funds together?

Is there anything else to add? please explain.

Do you have any other key informants in mind to introduce for interviewing?
